# Snail and SIP1 increase cancer invasion by upregulating MMP family in hepatocellular carcinoma cells

**DOI:** 10.1038/sj.bjc.6601685

**Published:** 2004-02-24

**Authors:** A Miyoshi, Y Kitajima, K Sumi, K Sato, A Hagiwara, Y Koga, K Miyazaki

**Affiliations:** 1 Department of Surgery, Saga Medical School, Nabeshima 5-1-1, Saga 849-8501, Japan

**Keywords:** snail, SIP1, E-cadherin, invasion, hepatocellular carcinoma

## Abstract

Loss of E-cadherin (E-cad) triggers invasion, metastasis, and dedifferentiation in various epithelial carcinomas. Recently, it has been reported that two transcription factors, Snail and SIP1 (Smad interacting protein 1), directly repress transcription of the E-cad gene by binding E-box on E-cad promoter. Our aim is to solve the molecular mechanism of Snail and SIP1 in hepatocellular carcinoma (HCC). We first showed an inverse correlation between E-cad and Snail/SIP 1 expression among five HCC lines with different phenotypes. The result indicated that undifferentiated, but not differentiated type expressed Snail/SIP1. Then, we established transfectants stably expressing Snail and SIP1 in two differentiated cells with E-cad expression. Suppressed expression of E-cad, morphologic change into fibroblastoid feature, and remarkable acceleration of invasion activity were observed in the transfectants. In reverse transcription–polymerase chain reaction series of genes relating to motility and invasion, we demonstrated striking evidence that matrix metalloproteinase (MMP-1), MMP-2, MMP-7, and MT1-MMP expressions were strongly upregulated by Snail. On the other hand, MMP-1, MMP-2, and MT1-MMP expressions were enhanced by SIP1 transfection, however, the intensity was weaker than that in Snail transfection. In conclusion, Snail or SIP1 expression may be induced during HCC progression, where Snail/SIP1 directly represses E-cad gene transcription and activates cancer invasion via the upregulation of the MMP gene family.

E-cadherin (E-cad), one of the key cadherins, plays a major role in the establishment and maintenance of intercellular adhesion, cell polarity, and tissue architecture (Takeuchi, 1991; Gumbiner, 1996). Reduced expression of E-cad is frequently associated with dedifferentiation, invasion, lymph node or distant metastasis, and poor prognosis in various human malignancies (Jiang, 1996; Jiao *et al*, 2001; Mukai *et al*, 2001; Tanaka *et al*, 2002). Several reports have demonstrated that in cases of hepatocellular carcinoma (HCC), E-cad expression significantly correlated with histological grade, vascular invasion, intrahepatic metastasis, and poor prognosis (Kozyraki *et al*, 1996; Endo *et al*, 2000). Thus, the molecular resolution of E-cad loss in HCC is of interest as new findings into the process of cancer dedifferentiation leading to invasion and metastasis development.

Recently, zinc-finger transcription factors Snail and SIP1 (Smad interacting protein 1) have been described to directly repress transcription of the E-cad gene by binding to the E-boxes (CACCTG sequence) on the E-cad promoter (Batlle *et al*, 2000; Comijn *et al*, 2001). Snail gene has been isolated in Drosophila embryos. During the embryonic development, Snail has been implicated in the triggering of epithelial–mesenchymal transition (EMT) in the precursors of the mesoderm and neural crest (Hemavathy *et al*, 2000; Carver *et al*, 2001; Nieto, 2001).

Another zinc-finger protein SIP1 is a novel member of the *δ*-crystallin enhancer binding factor 1 family, which had been characterised as a DNA-binding transcriptional repressor. Smad interacting protein 1 has been identified as a protein binding to the mad homology domain 2 domain of Smad1, which plays a critical role in TGF-*β* signalling and the bone morphogenetic protein (BMP) pathway (Remacle *et al*, 1999; Grusven *et al*, 2001). The SIP1 protein is also known to repress E-cad transcription by binding to the 5′-CACCT sequences on the promoter (Comijn *et al*, 2001).

Several reports have implicated Snail and SIP1 in not only E-cad repression, but acceleration of cancer invasion (Batlle *et al*, 2000; Cano *et al*, 2000; Cheng *et al*, 2001; Yokoyama *et al*, 2001; Blanco *et al*, 2002). However the precise mechanism, how Snail or SIP1 increases cancer invasion, remains to be unknown. We have currently reported an inverse correlation between E-cad and Snail expressions in HCC cell lines, in which differentiated cells expressed E-cad but not Snail whereas undifferentiated cells expressed Snail but not E-cad (Jiao *et al*, 2002).

In this study, we aimed to clarify whether the expression of Snail or SIP1 not only represses E-cad expression, but also affects cellular properties such as morphology, proliferation, and invasion. We finally attempted to isolate genes involving in cancer invasion accelerated by Snail or SIP1.

## MATERIALS AND METHODS

### Cells and cell culture

Five established HCC cell lines (HepG2, Huh-7, HLF, Changliver, and Hul-1) (Chang, 1954; Doi *et al*, 1975; Katsuta *et al*, 1980; Knowles *et al*, 1980; Nakabayashi *et al*, 1982) were purchased from RIKEN Cell Bank (Ibaragi, Japan) for use in this study. Cells were cultured in Williams E medium (Sigma, St Louis, MO, USA) supplemented with 10% heat-inactivated fetal bovine serum (Sigma, St Louis, MO, USA) and 100 *μ*g ml^−1^ kanamycin (Meiji, Tokyo, Japan), and incubated at 37°C under 5% CO_2_ in a humidified atmosphere. In the previous study, we defined HepG2 as differentiated type, but HLF, Changliver, and Hul-1 as undifferentiated type by histopathological analysis of their implanted tumours (Jiao *et al*, 2002). Huh-7 cells were established from a well-differentiated HCC tissue (Nakabayashi *et al*, 1982), and confirmed as differentiated type by the histopathological analysis of the implanted tumours.

### Reverse transcription–polymerase chain reaction

Total RNA was isolated from each cell line using Isogen (Nippongene, Toyama, Japan). Reverse transcription–polymerase chain reaction (RT–PCR) amplification was carried out using a commercial RNA LA PCR kit (AMV) version 1.1 (Takara Biochemicals, Shiga, Japan). The RNA samples (1 *μ*g) were converted into cDNA using random primers and reverse transcriptase. Amplification by PCR was carried out according to the manufacturer's instructions. The thermal cycles were: denaturing at 94°C for 30 s, annealing at 60°C for 30 s, and extension at 72°C for 90 s. The cDNA was amplified for 28 cycles. The primer pairs of E-cad, Snail, SIP1, and GAPDH were designed as follows: E-cad, forward 5′-TCC CAT CAG CTG CCC AGA AA-3′ and reverse 3′-TGA CTC CTG TGT TCC TGT TA-5′; human Snail, forward 5′-TTC TTC TGC GCT ACT GCT GCG-3′ and reverse 3′-GGG CAG GTA TGG AGA GGA AGA-5′; human SIP1, forward 5′-GGA AGA CAA GCT TCA TAT TGC-3′ and reverse 3′-ATG GCT GTG TCA CTG CGC TGA-5′; GAPDH, forward 5′-TGG TAT CGT GGA AGG ACT CAT GAC-3′ and reverse 3′-ATG CCA GTG AGC TTC CCG TTC AGC-5′. GAPDH was amplified in each sample as the internal marker. All reactions were repeated three times.

### Western blot analysis

Western blot was carried out as described previously (Jiao *et al*, 2002). Aliquots of each cell extract containing the same amount of protein (50 *μ*g for E-cad and *β*-actin) were resolved by 10% SDS–PAGE, and the separated extracts were electrophoretically transferred onto Hybond™ ECL™ membranes (Amersham Pharmacia Biotech, Buckinghamshire, UK) in a transfer buffer. The membrane was incubated with HECD-1 (a mouse monoclonal E-cad antibody, 1 *μ*g ml^−1^, Takara Biochemicals, Tokyo, Japan), and anti-*β*-actin (monoclonal anti-*β*-actin, clone AC-15, 1 : 1000 dilution; Sigma, St. Louis, MO, USA).

### Plasmids

The human E-cad promoter fragment spanning −218 to +47 at the transcription start site, which contained three E-box elements as previously described (Batlle *et al*, 2000), was amplified by PCR using genomic DNA from HepG2, along with KOD-Plus-DNA polymerase (Toyobo, Osaka, Japan) and oligonucleotides 5′-AGA ACC GTG CAG GTC CCA TAA-3′ and 3′-AAC TGA CTT CCG CAA GCT CAC-5′ corresponding to GenBank™ sequence L34545. The amplification product was purified, blunted, and connected upstream of luciferase in the pGL3 vector (Promega, Madison, WI, USA) (pGL3-E-cad). Direct sequencing confirmed that there was no mutation in the insert.

Full-length mouse Snail cDNA tagged with the haemagglutinin (HA) epitope at the C-terminus and inserted in the pCDNA3 expression vector was provided by Dr Antonio Garcia de Herreros (Unitat de Biologia Cellular y Molecular, Institut Municipal d’Investigacio Medica, Universitat Pompeu Fabra, Barcelona, Spain).

Full-length mouse SIP1 cDNA also tagged with the FLAG epitope at the N-terminus and cloned into the pCDNA3.1 expression vector was provided by Dr Kristin Verschueren (Department of Cell Growth, Differentiation, and Development VIB, and Laboratory of Molecular Biology, University of Leuven, Leuven, Belgium).

### Promoter reporter assays

For assessment of E-cad promoter repression by Snail or SIP1, 2×10^5^ cells of the HepG2 and Huh-7 cell lines were seeded. After incubation for 24 h, 1.0 or 1.5 *μ*g of pGL3-E-cad together with empty vector pcDNA-3.1, Snail, or SIP1 expression plasmids at 10, 50, and 100 ng were transiently introduced into the cell lines using LipofectAMINE™ reagent (Invitrogen Corp., Carlsbad, CA, USA). In all experiments, the total amount of transfected DNA was standardised with empty vector. At 48 h after transfection, luciferase activities were measured using Luciferase assay system (Promega, Madison, WI, USA) according to the manufacturer's instructions. Luciferase activities were normalised by *β*-galactosidase activity from cotransfected pSV*β*-galactosidase vector (pSV*β*) (Promega, Madison, WI, USA). Triplicates were systematically included and experiments were repeated at least three times.

### Establishment of stable transfectants

Snail and SIP1 stable transfections were introduced into HepG2 and Huh-7 cells using LipofectAMINE reagent (Invitrogen Corp., Carlsbad, CA, USA). At 24 h after seeding, 2×10^5^ cells per 60-mm dish were transfected with 5 *μ*g of Snail or SIP1 expression vector. The cells were cultured under G418 stress at 200–800 *μ*g ml^−1^ concentration (Promega, Madison, WI, USA). After 2 weeks of incubation, individual G418-resistant colonies were subjected to subcloning using a cloning ring (Iwaki, Chiba, Japan). As a control, empty pcDNA3.1 vector (Promega, Madison, WI, USA) was transfected into HepG2 and Huh-7 cells, and the established cells were used as control transfectants. Expressions of human E-cad, mouse Snail, and mouse SIP1 mRNA in the various clones were confirmed by RT–PCR. The primer pairs of Snail and SIP1 were designed as follows: mouse Snail, forward 5′-TTG TAA CAA GGA GTA CCT CAG-3′ and reverse 3′-GCA GCC AGA CTC TTG GTG CTT-5′; mouse SIP1, forward 5′-AGT CCA ATG CAG CAC TTA GGT-3′ and reverse 3′-TTC ATG CTG ATG CAG GGG AAT-5′. Expression of human E-cad, mouse Snail, and mouse SIP1-proteins was assessed by Western blot analysis. The following primary antibodies were used: E-cad, HECD-1 (a mouse monoclonal E-cad antibody, 1 *μ*g ml^−1^, Takara Biochemicals, Tokyo, Japan); mouse Snail, anti-HA monoclonal antibody (clone 3F10, 2.5 *μ*g ml^−1^, Sigma, St Louis, MO, USA); mouse SIP1, anti-FLAG polyclonal antibody (1 : 2000 dilution, Sigma, St Louis, MO, USA).

### Cell proliferation assay

Cell proliferation was analysed by the MTT proliferation method. In brief, 1×10^4^ cells well^−1^ were seeded in triplicate onto 96-well plates and incubated at 37°C under 5% CO_2_ in a humidified atmosphere. After 24 h, viable cell numbers were measured in triplicate every day for 4 days using the CellTiter96™ Non-Radioactive Cell Proliferation Assay Kit (Promega, Madison, WI, USA). The proliferation curves were constructed by calculating the mean value of optical density measurements at 570 nm using a 96-well plate reader.

### Transwell invasion assay

*In vitro* invasion activities through a gel matrix (Matrigel; Beckton Dickinson, Franklin Lakes, NJ, USA) were examined in 24-well plates as previously described with slight modification (Kohya *et al*, 2003). In brief, 6.5-mm diameter polycarbonate filters (8 *μ*m pore size) of the Falcon Transwell™ chemotaxis chambers (Beckton Dickinson, Franklin Lakes, NJ, USA) were coated with 50 *μ*l (0.2 mg ml^−1^) of Matrigel in cold W/E medium and dried overnight. Suspensions of 5×10^5^ cells in 200 *μ*l of complete W/E medium were plated on the upper compartment of the chamber. The MRC-5 conditioned medium (800 *μ*l) was placed in the lower compartment of the chamber to serve as the source of chemoattractants. After 48 h, noninvasive cells on the upper surface of the filters were removed completely by wiping the filter surface with a cotton swab. Viable invasive cells, which adhere to the lower surface of the filter, were fixed using 70% ethanol and the nuclei were stained using haematoxylin. Then, the number of invaded cells was counted. These experiments were carried out in triplicate and independently repeated at least three times.

### RT–PCR of candidate genes for motility, invasion, and mesenchymal molecules

The RT–PCR analysis was performed using 1 *μ*g of total RNA isolated from control, Snail, and SIP1transfectant cells. Primer sets and estimated lengths of PCR products for the cDNA amplification of candidate factor genes relating to motility, invasion activity, and mesenchymal markers are listed in Table 1

**Table 1:** PCR primers

**Gene**	**Sequence**	**Fragment size (bp)**
*Motility candidate genes*		
HGF	5′-GCCTGAAAGATATCCCGACA	525
	3′-TTCCATGTTCTTGTCCCACA	
c-met	5′-ACCTTGGTGCAGAGGAGCAAT	313
	3′-TAGAGCCATGTTGATGTTATC	
mts1	5′-TTTGATCCTGACTGCTGTCATGGC	340
	3′-AGAGGAGTTTTCATTTCTTCCTGG	
RhoA	5′-ATGCTTGCTCATAGTCTTCAG	483
	3′-CAGAGCAGCTCTCGTAGCCAT	
nm23	5′-AAGCAGCTGGAAGGAACC	497
	3′-CAATGTGGTCTGCCCTCC	
CD44	5′-CAACTCCATCTGTGCAGCAAA	307
	3′-GTAACCTCCTGAAGTGCTGCTC	
Integrin*β*1	5′-CTGAAAGACAAGTATGTTGAG	584
	3′-GAATGTGACTAGTGTGAAACA	
Integrin*β*4	5′-GCGACTATGAGATGAAGGTG	707
	3′-GTGAGTTGTAGTCCCGTGTG	
Integrin*α*4	5′-CCCCTACAACGTGGACACTGA	310
	3′-GCTGTCTGGAAAGTGTGACCC	
*Invasion candidate genes*		
MMP-1	5′-TCCAAGCCATATATGGACGTT	255
	3′-ACTTCATCTCTGTCGGCAAAT	
MMP-2	5′-GGCCCTGTCACTCCTGAGAT	474
	3′-GGCATCCAGGTTATCGGGGA	
MMP-3	5′-TATCACTCACTCACAGACCTG	334
	3′-TCCTTGCTAGTAACTTCATAT	
MMP-7	5′-ATGGGGAACTGCTGACATCAT	153
	3′-CCAGCGTTCATCCTCATCGAA	
MMP-9	5′-CAACATCACCTATTGGATCC	482
	3′-CGGGTGTAGAGTCTCTCGCT	
TIMP-2	5′-GGCGTTTTGCAATGCAGATGTAG	497
	3′-CACAGGAGCCGTCACTTCTCTTG	
MT1-MMP	5′-CTAAGACCTTGGGAGGAAAAC	192
	3′-AAGCCCCATCCAAGGCTAACA	
uPA	5′-AAGAGTGCATGGTGCATGAC	320
	3′-CTTGCGTGTTGGAGTTAAGC	
*Mesenchymal marker*		
Vimentin	5′-ACGCCATCAACACCGAGTTCA	383
	3′-GTGCCAGAGACGCATTGTCAA	
Fibronectin	5′-AGGAAGCCGAGGTTTTAACTG	314
	3′-TCAGCTATGGGCTTGCAGGTC	

HGF=hepatocyte growth factor; MMP=matrix metalloproteinase; UPA=urokinase plasminogen activator.

. Each experiment was carried out in triplicate.

### Quantitative RT–PCR

To quantitatively estimate the mRNA expression of several candidate genes, PCR was performed on a Light-Cycler instrument system (Roche, Mannheim, Germany) using the Light-Cycler-FastStart DNA Master SYBR green I Kit (Roche, Mannheim, Germany) according to the manufacturer's instructions. The PCR product was quantified using GAPDH as a standard, and the relative expression rate was calculated by comparisons with the quantitative value of the control transfectant. These experiments were carried out in triplicate and independently repeated at least three times.

### Statistical analysis

Results of invasion assay and quantitative RT–PCR were statistically analysed using Student's *t*-test to compare between control and Snail/SIP1 transfectant. *P*-value less than 0.05 was considered statistically significant.

## RESULTS

### Relationship between Snail, SIP1, and E-cad in five HCC cell lines

In this study, five HCC cell lines (HepG2, Huh-7, HLF, Changliver, and Hul-1) were analysed.

**Figure 1:**
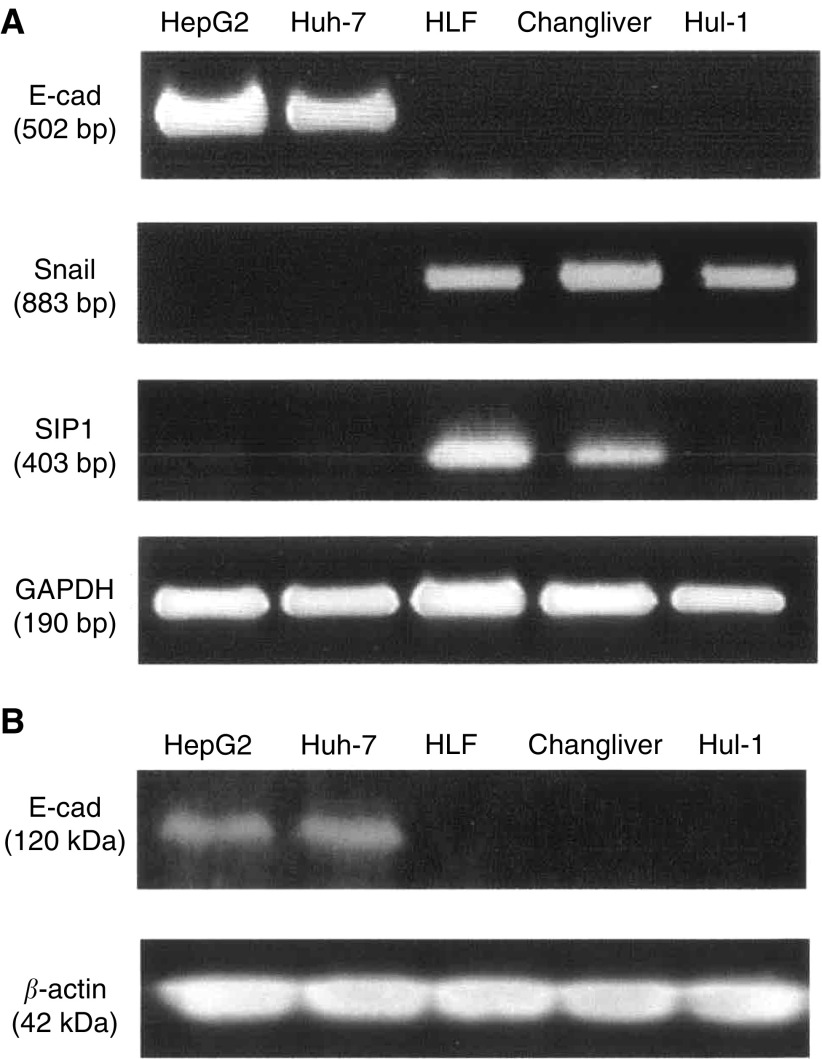
E-cadherin, Snail, and SIP1expressions were analysed and compared among five HCC cell lines by RT–PCR and the Western blot method. (**A**) RT–PCR: differentiated cell lines HepG2 and Huh-7 expressed E-cad but not Snail or SIP1. Undifferentiated cell lines HLF and Changliver expressed Snail and SIP1 but not E-cad. The undifferentiated cell line Hul-1 expressed Snail but not SIP1 or E-cad. GAPDH was used as an internal marker. (**B**) Western blot: E-cad protein was expressed in HepG2 and Huh-7 cell lines. *β*-actin protein levels were used to normalise the Western blot reactions.

In RT–PCR analysis, E-cad mRNA expression was found in differentiated cell lines HepG2 and Huh-7 cells, but not in undifferentiated cell lines HLF, Changliver, and Hul-1. Conversely, Snail and SIP1 mRNA expressions were detected in HLF and Changliver cell lines but not HepG2 and Huh-7 cell lines (Figure 1A

). These results showed an inverse correlation between Snail, SIP1, and E-cad among the four HCC cell lines HepG2, Huh-7, HLF, and Changliver. The fifth cell line Hul-1 expressed Snail but not E-cad or SIP1. We also analysed E-cad protein expression in these cell lines by the Western blot method. The E-cad protein was detected in HepG2 and Huh-7 cell lines but not in the HLF, Changliver, and Hul-1 cell lines (Figure 1B).

### Inhibition of E-cad promoter activity by Snail and SIP1

**Figure 2:**
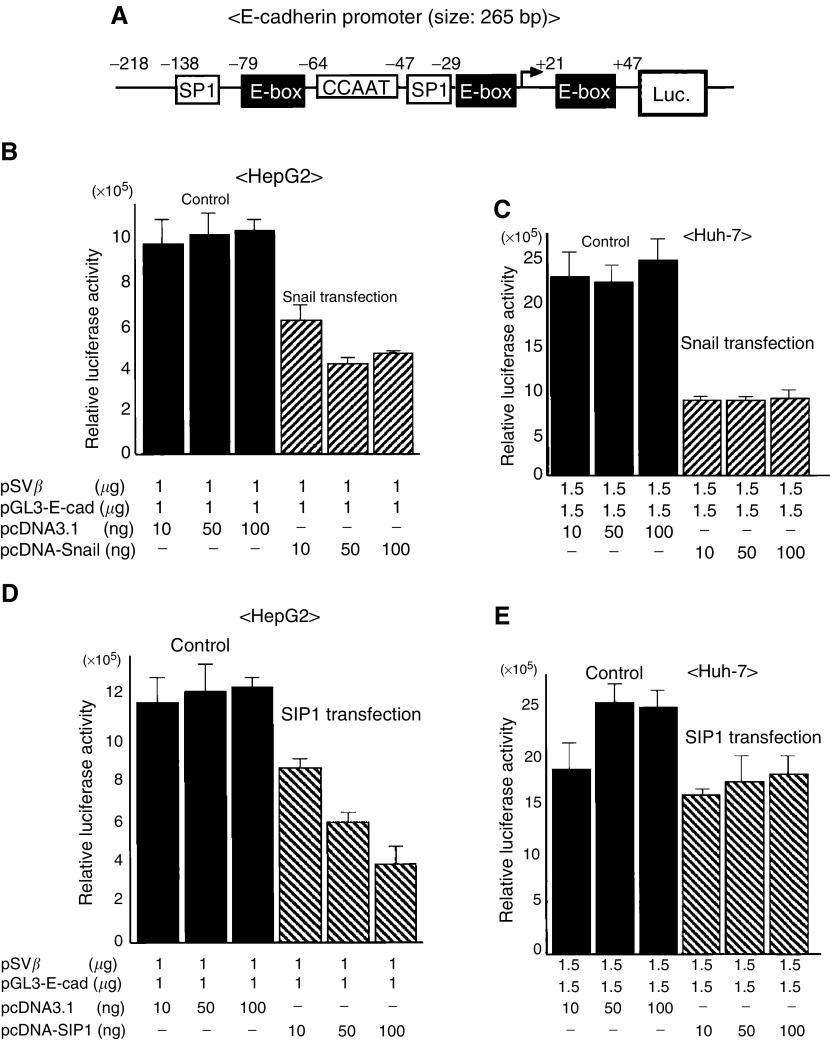
(**A**) The human E-cad promoter fragment spanning −218 to +47 at the transcription start site, which contained three E-boxes elements was amplified by PCR using genomic DNA from HepG2. The promoter fragment was ligated upstream from the luciferase gene in the pGL3-basic plasmid (pGL3-E-cad). The repression effect on E-cad promoter activity by Snail and SIP1 in HepG2 and Huh-7 cell lines. Cells were cotransfected with 1 or 1.5 *μ*g of pGL3-E-cad and various amounts of Snail or SIP1 expression plasmid. Luciferase activities were normalised by *β*-galactosidase activities and shown by mean value±standard deviation of the triplicate measurements. (**B, C**) Snail repressed E-cad promoter activity. Both cell lines transfected with Snail expression plasmid showed luciferase activity at about one-third compared with control. (**D**) In the HepG2 cell line, the promoter activity was clearly repressed by SIP1 expression in a dose-dependent manner and the minimal activity was about 30% of the control activity. (**E**) In Huh-7 cells, the promoter activity in SIP1 transfection showed about 70% activity of control transfection.

We examined the E-cad promoter activity in these cell lines using a luciferase reporter plasmid connected to a proximal E-cad promoter fragment upstream of luciferase gene. As shown in Figure 2

, the isolated promoter fragment contained three E-boxes, which are defined as the binding sites of Snail and SIP1 (Batlle *et al*, 2000; Comijn *et al*, 2001). Using the reporter plasmid, we investigated whether Snail and SIP1 repress E-cad promoter activity in E-cad-expressing HepG2 and Huh-7 cells. In both cell lines, Snail transfection reduced the luciferase activity to one-third, compared with control transfection (Figure 2B, C). Smad interacting protein 1 also decreased E-cad promoter activity; however, the repressive effect in Huh-7 cells was less than that in HepG2 cells (Figure 2D, E). The promoter activity in HepG2 was clearly repressed by SIP1 expression in a dose-dependent manner and maximum effect was about 30% of the control activity (Figure 2D). In Huh-7 cells, SIP1 transfection resulted in about 70% activity of control transfection (Figure 2E).

### Effects on the cell morphology, proliferation, and invasion by Snail and SIP1 expression

**Figure 3:**
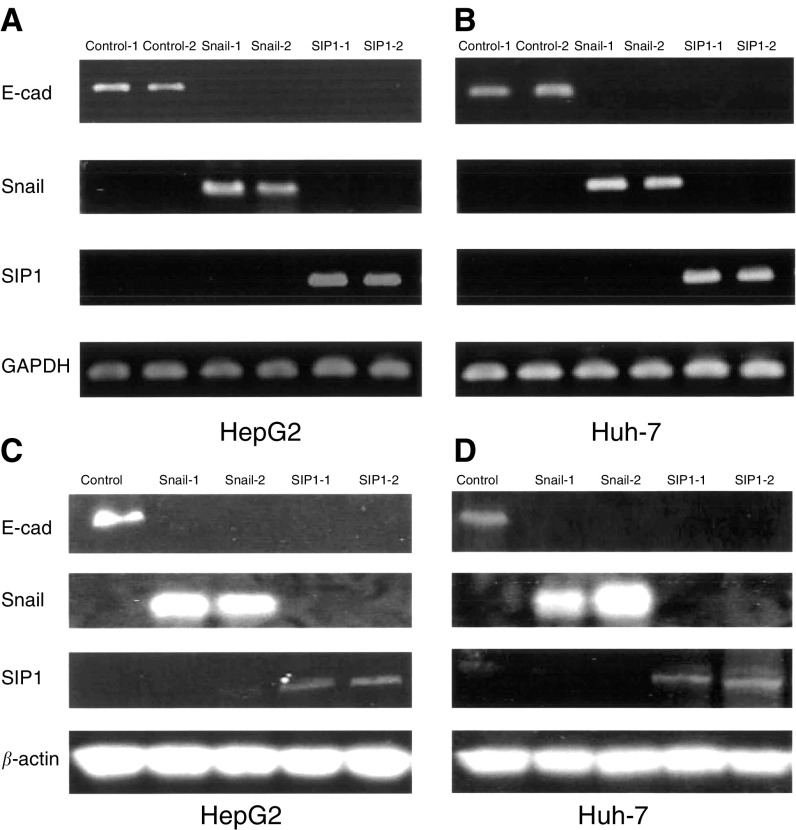
Exogenous expression of Snail and SIP1 repressed E-cad mRNA and protein. HepG2 and Huh-7 cell lines were transfected with expression vector harbouring cDNA encoding HA epitope-tagged full-length mouse Snail (Snail-1, -2), FLAG-tagged full-length mouse SIP1 (SIP1-1, -2). Individual cell clones were isolated after selection by G-418. (**A, B**) E-cadherin, Snail, SIP1, and GAPDH expressions were determined by RT-PCR. GAPDH was used as an internal marker. (**C, D**) Western blot was carried out to examine E-cad, Snail, SIP1, and *β*-actin protein expressions. *β*-actin protein levels were used to normalise the Western blot reactions.

**Figure 4:**
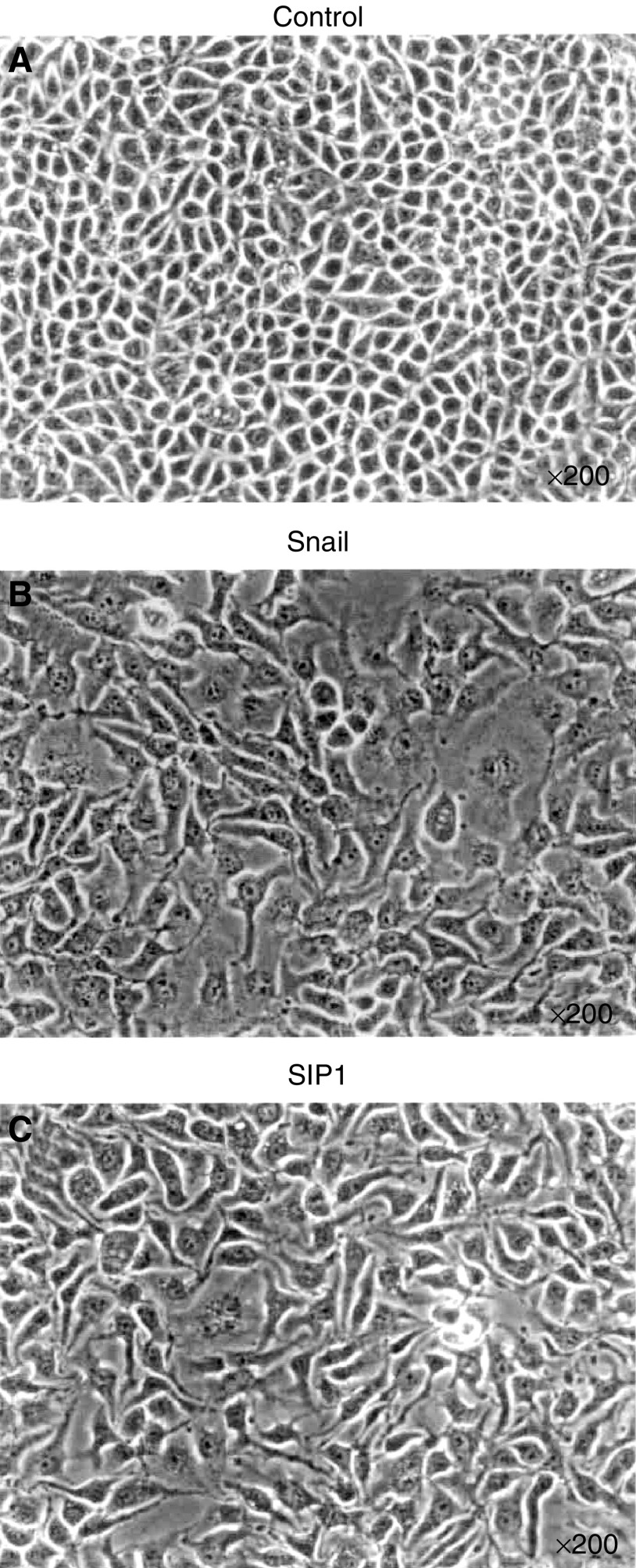
Microscopic features of stable transfectant in HepG2 cells. (**A**) control; (**B**) Snail; (**C**) SIP1. Photographs were taken at a magnification of ×200. The morphologic alteration to fibroblastoid features was observed in Snail and SIP1 transfectants compared with control cells.

We established stable transfectants that expressed Snail or SIP1 using each expression plasmid in HepG2 and Huh-7 cell lines. Snail and SIP1 transfectants showed undetectable expression of E-cad mRNA in both cell lines, compared with the control transfectant (Figure 3A, B

). Consequently, expression of E-cad protein was repressed by Snail and SIP1 (Figure 3C, D). Microscopic study showed that introduction of Snail and SIP1 plasmid in HepG2 changed the morphologic features to a flattened and fibroblast-like phenotype, compared with the control transfection (Figure 4

).

**Figure 5:**
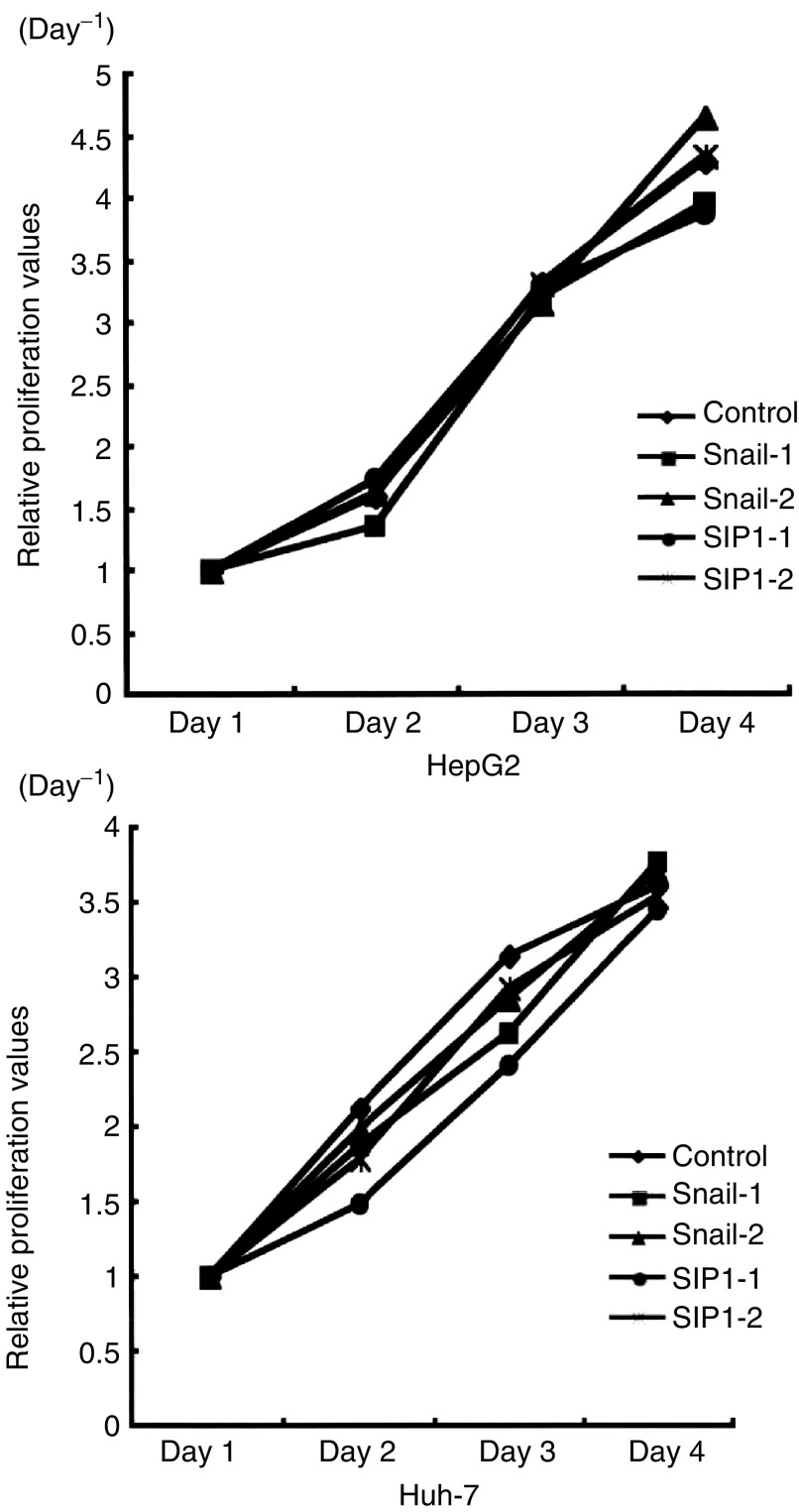
MTT proliferation assay was carried out using each transfectant cell. Cells (1×10^4^) were plated on 96-well plates and after 24 h; MTT activities were measured in triplicate on days 1, 2, 3, and 4. The proliferation curves were illustrated by plotting the average of triplicated values calculated by optical density measurements at 570 nm in a 96-well plate reader. Relative proliferation values on days 2–4 were shown as ratio to OD570 nm on day 1. All transfectants in HepG2 and Huh-7 cells showed similar growth curves and there was no significant difference.

**Figure 6:**
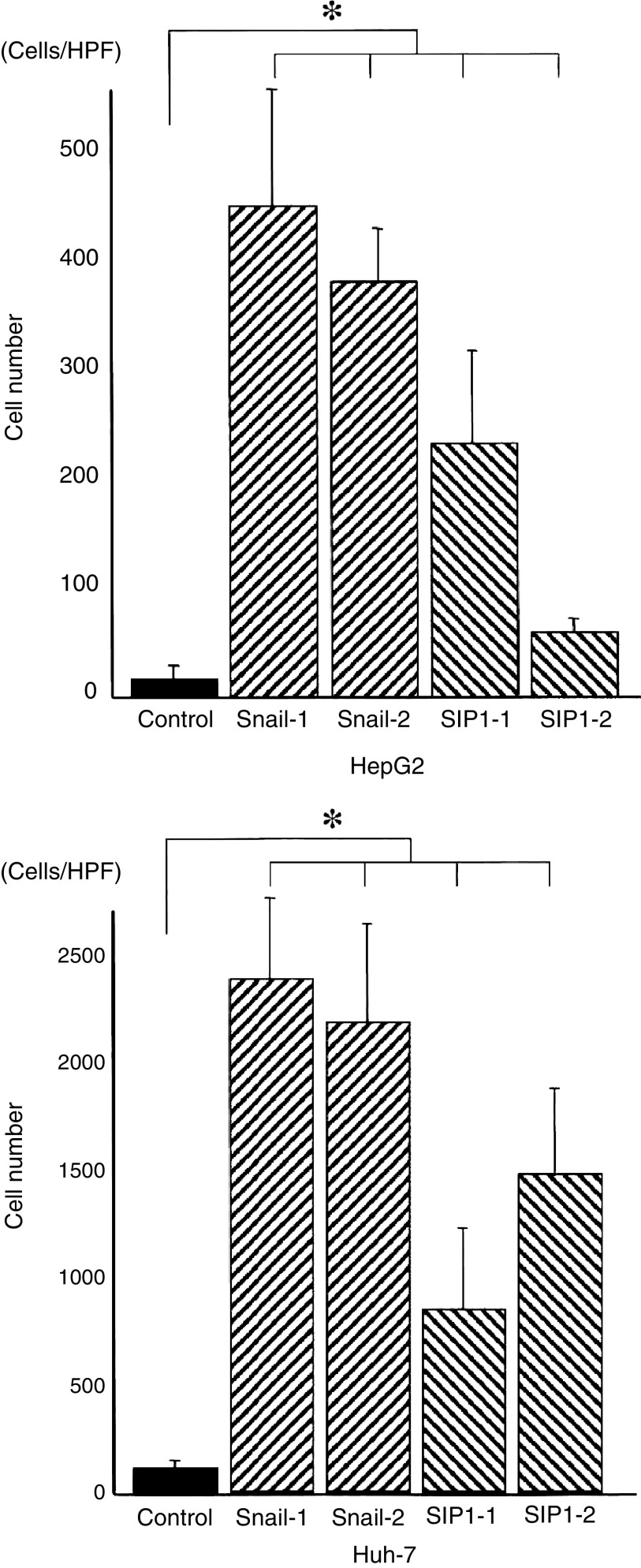
Invasion ability of each cell was analysed in the transwell invasion assay. Data are presented as mean value±standard deviation of the triplicate measurements. Snail and SIP1 transfectant cells showed significantly higher invasive properties compared with control cells (^*^P<0.05). In particular, Snail transfectants showed stronger invasive properties than SIP1 transfectants.

Using Snail and SIP1 transfectants in HepG2 and Huh-7 cell lines, we carried out MTT proliferation assays and *in vitro* invasion assays. As shown in Figure 5

, there was no change in cell proliferation between Snail/SIP1 transfectants and control cells (Figure 5). On the other hand, the invasion activity of Snail or SIP1 transfectant cells was significantly increased compared with that of the control cells (Figure 6

). Higher invasion activity was showed in Snail than SIP1 transfectant cells.

### Examination of candidate genes relating to cell motility, invasion, and mesenchymal transition affected by Snail and SIP1

In order to identify gene relating to invasion activated by Snail or SIP1, RT–PCR analysis using a primer set (Table 1) for each various candidate gene was carried out. The amplification products were evaluated in Snail-1 and SIP1-1 transfectants that showed the strongest activities in invasion assay.

For motility genes, there were no apparent differences in the expression of hepatocyte growth factor, c-met, mts1, RhoA, nm23, integrin*β*4, and integrin *α*4 among control, Snail-1, and SIP1-1 transfectant cells. Expression of the integrin*β*1 gene was not observed in any cell (data not shown).

**Figure 7:**
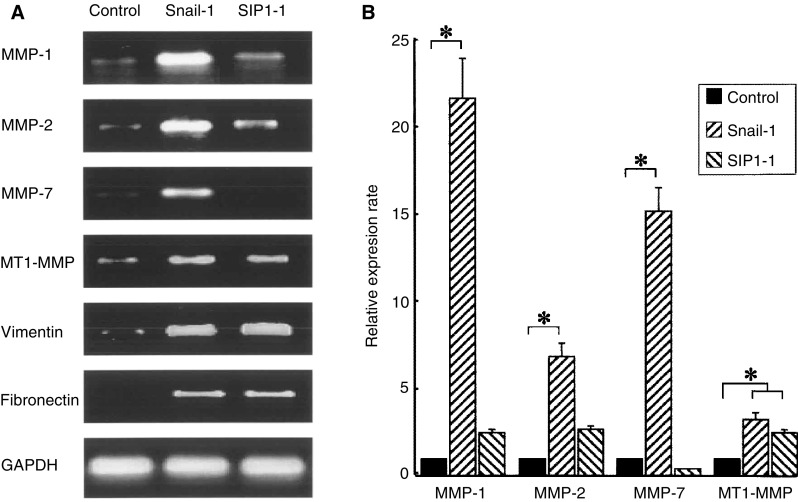
RT–PCR was performed using Snail and SIP1 transfectants in HepG2. (**A**) In candidate genes relating to cancer invasion, MMP-1, MMP-2, MMP-7 and MT1-MMP expressions were upregulated by Snail. Matrix metalloproteinase-1, MMP-2, and MT1-MMP were increased by SIP1 transfection. Genes for mesenchymal marker, vimentin, and fibronectin showed higher expression in Snail and SIP1 transfectants, compared with the control cells. (**B**) In quantitative RT–PCR analysis using Light-Cycler system, the expression amount of MMP genes was calculated by quantitative value with GAPDH. Relative expression rate for each MMP family was indicated as ratio to values with control. Mean value±standard deviation of the triplicate measurements was calculated and illustrated in the histogram of the right panel. Expression of MMP-1 was 22-fold higher in Snail-1 (^*^P<0.05) and 2.5-fold higher in SIP1-1 than control. Expression of MMP-2 was 8.7-fold higher in Snail-1 (^*^P<0.05) and 3.2-fold higher in SIP1-1. Expression of MMP-7 was 15.8-fold in Snail-1 (^*^P<0.05), MT1-MMP was 3.4-fold higher in Snail-1 (^*^P<0.05) and 2.7-fold higher in SIP1-1 (^*^P<0.05).

Among invasion candidate genes, expressions of matrix metalloproteinase (MMP-1), MMP-2, MMP-7 and MT1-MMP were remarkably elevated in Snail-1 compared with control transfectants. Expressions of MMP-1, MMP-2, and MT1-MMP were also upregulated in SIP1-1 transfectant (Figure 7A

). On the contrary, there were no differences in the expression of tissue inhibitor of metalloproteinase and urokinase plasminogen activator. No expression of MMP-3 or MMP-9 was found in any transfectant in HepG2 cells (data not shown).

Genes for mesenchymal markers such as vimentin and fibronectin indicated higher expression in Snail and SIP1 transfectants compared with control cells (Figure 7A).

### Quantification of mRNA expression of MMP gene family

Conventional RT–PCR revealed that Snail and SIP1 transfection influenced expression of MMP gene family. To evaluate precise differences in MMP-1, MMP-2, MMP-7, and MT1-MMP expression among control, Snail-1, and SIP1-1 transfectant, quantitative RT–PCR was carried out with a Light-Cycler-assisted approach using the SYBR green I system. The result was shown in Figure 7B. In comparison with control transfectant, expression of MMP-1 was 22-fold higher in Snail-1 and 2.5-fold higher in SIP1-1. Expression of MMP-2 was 8.7-fold higher in Snail-1 and 3.2-fold higher in SIP1-1. Expression of MMP-7 was 15.8-fold in Snail-1; MT1-MMP was 3.4-fold higher in Snail-1 and 2.7-fold higher in SIP1-1. Statistical analysis also revealed MMP-1, MMP-2, MMP-7, and MT1-MMP expressions were significantly upregulated by Snail, whereas only MT1-MMP expression was upregulated by SIP1 (^*^P<0.05).

These results indicate that MMP gene family was upregulated more strongly by Snail than SIP1 transfection.

## DISCUSSION

Several reports have demonstrated that the downregulation of E-cad in HCC is significantly associated with large tumour size, low grade of histological differentiation, capsular and vascular invasion, early recurrence, intrahepatic metastasis, and poor prognosis (Kozyraki *et al*, 1996; Endo *et al*, 2000). Two mechanisms in the downregulation of E-cad expression in HCC have been demonstrated; one is by hypermethylation of the E-cad promoter and the other is by loss of heterozygosity (LOH) of the E-cad gene (Kanai *et al*, 1997; Matsumura *et al*, 2001; Wei *et al*, 2002). In this experiment, we have focused on another mechanism of E-cad repression by zinc finger transcription factors Snail and SIP1 in HCC cell lines, since Snail and SIP1 have been described to not only repress E-cad expression, but also contribute to cellular dedifferentiation and cancer invasion (Batlle *et al*, 2000; Cano *et al*, 2000; Comijn *et al*, 2001; Yokoyama *et al*, 2001).

An inverse correlation between E-cad and Snail has been reported in a panel of epithelial and dedifferentiated cells derived from carcinomas in various etiologies, including oral squamous carcinoma, cancers of the breast, pancreas, colon, and bladder, melanoma, and HCC (Batlle *et al*, 2000; Cano *et al*, 2000; Cheng *et al*, 2001; Yokoyama *et al*, 2001; Blanco *et al*, 2002; Jiao *et al*, 2002).

We showed that differentiated HCC cell lines HepG2, and Huh-7 expressed E-cad whereas the undifferentiated cell lines HLF, Changliver, and Hul-1 expressed Snail, SIP1, or both. These results suggested that during the histological change from differentiation to undifferentiation, Snail and SIP1 expressions are induced, and caused the repression of E-cad expression. In the reporter plasmid with the E-cad core promoter containing three E-boxes, both Snail and SIP1 repressed luciferase activity driven from the reporter plasmid in HepG2 and Huh-7 cells. These results support the previous findings that Snail and SIP1 directly repressed E-cad promoter activity by binding to overlapping three E-box CACCTG sequences (Batlle *et al*, 2000; Comijn *et al*, 2001). In the experiment using a stable transfectant of Snail or SIP1 in HepG2 and Huh-7 cells, E-cad expression was dramatically repressed, supporting the previous reports of E-cad repression mechanism by Snail and SIP1.

To elucidate the hypothesis that Snail or SIP1 expression causes dedifferentiation and increases cancer invasion, alteration in morphologic features and invasion activity were assessed using the Snail and SIP1 transfectants. As a result, phenotypic alteration to the fibroblastoid features was found in the transfectant series of HepG2. It was reported that hepatocellular EMT correlated with both dedifferentiation and invasion (Gotzmann *et al*, 2002). Therefore this *in vitro* EMT phenomenon may reflect histological change from differentiated to undifferentiated type during HCC progression. The invasive activity in Snail and SIP1 transfectant from HepG2 and Huh-7 cells was significantly increased compared with control transfectant. A stronger effect in increasing invasion was found in Snail, compared with SIP1 transfection in HepG2 as well as Huh-7.

Snail and SIP1 are classified into a family of zinc-finger transcription factor and exert promoter silencing to partly overlapping promoter sequences CACCTG located on E-cad promoter (Comijn *et al*, 2001). However, each protein structurally differs: Snail contains four or five zinc-finger domains at the C-terminal end, while SIP1 is characterised by a homodomain flanked by an N-terminal that contains four zinc-fingers and a C-terminal cluster that contains three zinc-fingers (Remacle *et al*, 1999; Hemavathy *et al*, 2000). These reports prompted a hypothesis that Snail and SIP1 may distinctly regulate other genes, except for E-cad via different binding property to E-box on some gene promoter.

We attempted to identify genes relating to dedifferentiation, cell motility, and invasion, which are induced by the expression of Snail or SIP1. RT–PCR analysis of several candidate genes was carried out using Snail and SIP1 transfectants in HepG2 cells. Expression of vimentin and fibronectin genes relating to EMT was dramatically increased in Snail as well as SIP1 transfectant. In candidate genes relating to cancer motility and invasion, MMP gene family was upregulated by Snail or SIP1 expression in HepG2 cells. Snail transfection into HepG2 increases mRNA of MMP-1, MMP-2, MMP-7, and MT1-MMP, whereas SIP1 upregulated MMP-1, MMP-2, and MT1-MMP mRNA. We also quantified expressions of MMP gene family in Snail and SIP1 transfectant using Light-Cycler system. The candidate MMP genes were more strongly upregulated by Snail than SIP1, regardless of same expressions of EMT markers, vimentin, and fibronectin. We also confirmed that the amount of Snail mRNA in Snail-1 was not different from SIP1 mRNA in SIP1-1 (data not shown). Thus, the different intensities in MMP induction between Snail and SIP1 transfectant may contribute to the difference in the invasion activity, however, we did not precisely compare the protein levels between Snail and SIP1 among the transfectants established.

Matrix metalloproteinase family is known to play a key role in the tumour invasion of various human cancers including HCC (Nabeshima *et al*, 2002). It was reported that MMP-1 expression was associated with portal invasion (Okazaki *et al*, 1997). Increased expression of MMP-2, MMP-7, and MT1-MMP had a strong association with dedifferentiation, portal invasion, intrahepatic metastasis, and recurrence (Yamamoto *et al*, 1997; Harada *et al*, 1998; Maatta *et al*, 2000). Transcriptional regulation of MMP has been previously reported in several studies. Binding sites for ETS or AP-1 transcription factors are found in the promoters of MMP family and these transcription factors have been reported to regulate MMP gene expression (Westernmarck and Kahari, 1999; Singh *et al*, 2002). Yokoyama *et al* (2003) showed MMP-2 gene expression and the promoter activity was increased by Snail transfection. However, to our knowledge, the E-box CACCTG site, which is the binding site of Snail and SIP1, was not observed in the promoter region of the MMP gene family. Snail and SIP1 may not directly upregulate MMP transcription and further analysis is necessary to clarify how Snail or SIP1 activate the transcription of MMP family.

In this study, we demonstrated that Snail and SIP1 independently confer a repression of E-cad and increase the invasive activity in HCC cell lines. In particular, we found that cancer invasion activated by Snail and SIP1 may depend on induction of the MMP family. During HCC progression, histological alteration from well-to-poorly differentiated types is observed and this event triggers cancer invasion, intrahepatic metastasis, and causes poor prognosis of patients. Our results indicated that Snail and SIP1 might be crucial molecules to govern cellular function such as E-cad repression, acceleration of cancer invasion, and dedifferentiation during HCC progression. Therefore, molecular targeting therapy for Snail and SIP1 may lead to inhibition of vascular invasion, metastasis, and improvement of prognosis in patients with HCC.
